# The association of lipid-lowering therapy and blood pressure control among outpatients with hypertension at the Felege Hiwot Comprehensive Specialized Hospital, Northwest Ethiopia

**DOI:** 10.3389/fcvm.2023.1071338

**Published:** 2023-03-01

**Authors:** Rahel Belete Abebe, Sewnet Adem Kebede, Mequanent Kassa Birarra

**Affiliations:** ^1^Department of Clinical Pharmacy, School of Pharmacy, College of Medicine and Health Sciences, University of Gondar, Gondar, Ethiopia; ^2^Department of Epidemiology and Biostatistics, Institute of Public Health, College of Medicine and Health Sciences, University of Gondar, Gondar, Ethiopia

**Keywords:** statin therapy, hypertension, Ethiopia, blood pressure control, lipid-lowering therapy, statin

## Abstract

**Background:**

The lipid-lowering medications known as statins have been shown in controlled clinical trials to have pleiotropic properties, such as lowering blood pressure, in addition to lowering cholesterol levels. The purpose of this study was to see if there was a possible link between blood pressure control and statin therapy in outpatients with hypertension in a real clinical setting.

**Patients and methods:**

A retrospective comparative cohort study of 404 patients with hypertension was carried out. A systematic random sampling technique was used. For data entry, Epi-Data version 4.6 was used, and SPSS version 25 was used for further analysis. For group comparisons, chi-square and independent *t*-tests were computed. To determine the relationship between statin use and blood pressure control, a binary logistic regression model was employed. To declare statistical significance, a 95% confidence interval and a *P*-value of <0.05 were used.

**Results:**

Half of the study participants who were using a prescribed statin were assigned to the statin group, whereas the remaining participants who do not take statins were assigned to the control group. After 3 months of statin treatment, BP control to <130/80 mmHg was significantly greater (*P* = 0.022) in the statin group (52.5%) than in the control group (41.0%). The use of statins raises the likelihood of having blood pressure under control by 1.58 times when compared to statin non-users. After controlling for possible confounders, statin therapy still increased the odds of having controlled BP by a factor of 5.98 [OR = 5.98; 95% CI: 2.77–12.92].

**Conclusion:**

This study revealed that blood pressure control was higher among statin user hypertensive patients. Favorable effects of statin use were independently observed, even after correction for age, presence of dyslipidemia, and duration of antihypertensive therapy. Therefore, the importance of concomitantly added lipid-lowering drugs such as statins and their role in managing poor blood pressure control should be given due emphasis.

## Introduction

Long-term complications including heart failure, myocardial infarction, kidney disease, and stroke are significantly increased when blood pressure is uncontrolled (1, 2). Despite the high prevalence of hypertension, only a minority of patients in developing nations receive treatment and achieve blood pressure control (3). Hypertension is a highly prevalent and poorly controlled chronic condition in Ethiopia (4, 5). Lowering intensive blood pressure (BP) to achieve a target BP of <130/80 mmHg is advantageous in decreasing cardiovascular outcomes (6). The high prevalence of hypertension, continuing evidence that it is undertreated, and a growing awareness of the adverse consequences of inadequately managed and poorly controlled hypertension lead studies into exploring other management options for poorly controlled blood pressure.

The advantages of BP medications for the prevention of cardiovascular events are widely known. However, the degree to which the effect of BP lowering treatments differs by the presence of different drug classes to manage comorbidities in patients with hypertension is less clear. Previous clinical studies have shown that some supplements and medications, including Silymarin, omega-3 fatty acids, Daflon, and febuxostat, have an effect on hypertensive markers, blood glucose control, blood pressure control, and controlling different biomarkers, all of which are associated with an increased risk of cardiovascular disease (7–10). Statins are one of the commonly prescribed drug classes for patients with hypertension with different comorbidities. They inhibit 3-hydroxy-3-methylglutaryl-coenzyme A (HMGCoA), a rate-limiting enzyme in cholesterol synthesis, to reduce cholesterol production in hepatocytes (11). Statins are recognized to improve cardiovascular protection on top of their lipid-lowering ability; in addition to this, they also have non-lipid-lowering pleiotropic effects (12). The potential mechanisms that could be involved in this effect include the increase in the endothelial synthesis of nitric oxide, the downregulation of the angiotensin II-type 1 receptor, and the reduction of the vasoconstrictor endothelin-1 level (12, 13).

Due to reports from different studies suggesting the potential role of statin therapy in BP control and reduction, statin use has sparked attention in the field of hypertension (14–17). Although statins are effective in hypertensive animal models and controlled clinical trials, it is important to see if a similar effect is observed in real clinical practice (18–21). However, so far, no studies have been conducted in Ethiopia to investigate the association of statins with blood pressure control. As a result, the purpose of this study was to see if there was a possible link between statin therapy and blood pressure control among patients with hypertension in a real-world clinical setting.

## Materials and methods

### Study design and area

A retrospective comparative cohort study was employed at the chronic outpatient clinic of the Felege Hiwot Comprehensive Specialized Hospital from 22 June to 21 August 2021. The hospital is located in Bahir Dar, a city in northwest Ethiopia, 490 km from Addis Ababa, Ethiopia's capital city.

### Populations

#### Source population

All patients with hypertension who were receiving antihypertensive therapy and attending the Felege Hiwot Comprehensive Specialized Hospital's chronic follow-up units.

#### Study population

All adult patients with hypertension who met the inclusion criteria and were under follow-up at the Felege Hiwot Comprehensive Specialized Hospital during the time of data collection were included in the study.

### Eligibility criteria

#### Inclusion criteria

All patients with hypertension who were ≥18 years of age and on standard antihypertensive therapy for at least 6 months were included in the control group or the non-statin user group, whereas all patients with hypertension who were ≥18 years and on standard antihypertensive and concurrent statin treatments for at least 6 months before the start of the study were included in the study user group.

#### Exclusion criteria

Patients with hypertension who refused to participate at the time of data collection, who had mental health issues, or who were unable to communicate, and patients with incomplete medical reports were excluded.

### Sample size determination

The sample size was calculated by utilizing Epi info software. Because no similar study had been conducted in the study area, 50% of the prevalence of the outcome among the exposed group was considered. When determining the sample size, the following factors were taken into account: 5% for two-tailed type one error (Zα = 1.96) and 80% for the power of study, a two-sided 95% confidence interval (CI), and a 1:1 comparison group ratio. The highest sample size number was found with the Fleiss with CC method, and the calculated sample size was 366. For possible missed data and lost to follow-up, a 10% contingency was considered, and finally, the study enrolled 404 patients.

### Sampling technique and procedure

The study participants who met the inclusion criteria were chosen using a systematic random sampling technique. The “*K*” value for the sampling interval was derived as *K* = *N*/*n*, where “*N*” is the estimated number of average monthly follow-ups of patients with hypertension in the hospital which was 814 and “n” is the final sample size which was 404 (22). Since the data collection period was for 2 months, our N was 1,628 which gives a sampling interval of four.

### Study variables

#### Dependent variable

The dependent variable was blood pressure control.

#### Independent variables

Sociodemographic characteristics (sex, age, and place of residence), statin use, presence of comorbidities, alcohol drinking status, smoking status, level of physical activity, level of adherence to medications, duration of hypertensive treatment, presence of co-administered drugs, and type, amount, and frequency of antihypertensive drugs.

### Operational definitions

#### Hypertension

It is defined as having a systolic BP of ≥130 mmHg or a diastolic BP of ≥80 mmHg or self-reported ongoing utilization of antihypertensive therapy as per the American College of Cardiology and the American Heart Association (ACC/AHA) 2017 guideline criteria (23).

#### Treatment for hypertension

It is characterized as the current usage of antihypertensive therapy as reported on a chart by individuals who have been told that they had high BP by a doctor or other health professional.

#### Blood pressure control

As per the ACC/AHA 2017 guideline, adults with proven hypertension and no additional markers of elevated cardiovascular disease (CVD) risk or with known CVD or 10-year arteriosclerotic cardiovascular disease (ASCVD) event risk of 10% or greater are considered as having both SBP <130 mmHg and DBP <80 mmHg (23).

### Data collection procedure and quality control

#### Data collection tools and procedures

To collect the data, a pretested, structured, interviewer-administered questionnaire developed by reviewing several works of literature was used (14, 15, 20, 24–29).

Three BSC nurses were involved in the data collection, one as a supervisor and the others as data collectors. The purpose of the questionnaire was to obtain information on the sociodemographic characteristics, lifestyle behavior, and clinical characteristics of respondents and their level of BP control with the different components of BP control evaluations. Information about sociodemography and lifestyle behavior was gathered by face-to-face interviews, while clinical parameters including prescribed medicines, BP measurements, and other objective measurements were obtained from the patient's medical records.

Three consecutive systolic and diastolic BP measurements were taken from the patient's medical charts with 3 months gap between them. The baseline BP value was the one recorded just before starting to take antihypertensive treatment for the control group and before starting to take concurrent statin treatment for the statin user group. Then, after 3 and 6 months of taking only antihypertensive treatment and antihypertensive with statin treatment, two different SBP and DBP values were recorded from the patient's charts for the control group and the statin user group, respectively.

The participants' adherence was assessed using the seven-item Adherence to Refills and Medications Scale (ARMS-7), a self-reported validated measure of medication adherence that is a simplified version of the ARMS (28). Each item was designed for a response on a 4-point Likert scale with replies ranging from “none,” “some,” “most,” or “all” of the time, which were allocated values from 1 to 4, respectively. The total score of the ARMS seven-item version varies between 7 (best adherence) to 28 (worst adherence), and it can be dichotomized as 7 or >7. Any score >7 shows some degree of non-adherence or poor adherence while a score equal to 7 suggests optimal or good adherence.

A questionnaire based on the Diet History Questionnaire-NIH and customized for use in Ethiopian settings was used to collect self-reported data about food consumption habits, with a four-item Likert scale (every day, frequently, rarely, or never). The primary goal of the questionnaire was to assess the overall eating habits in terms of frequency (daily, weekly, and monthly) and amount of fruits, vegetables, meat, and beverages, such as alcohol consumption.

Physical activity was measured by assessing how many minutes and days a patient spent exercising on a daily and weekly basis, respectively. Participants were considered physically active if they could carry out physical activities for at least 30 min per day for at least 5 days per week (≥150 min per week) and insufficient physical activity was defined as <30 min per day of moderate-intensity exercise for <5 days per week; otherwise, they were categorized as physically inactive if they did not exercise for at least 10 min per day (30).

Alcohol intake was considered excessive in this study if men consumed >10 glasses of wine, >21 Birille of Tej, >21 can/Tassa of Tella, >21 shot of spirit/Areki Melekia, or >14 standard drinks of beer per day, and if women consumed >7 glasses of wine, >10 Birille of Tej, >10 can/Tassa of Tella, >10 shot of spirit/Areki, or >7 standards drink of beer per day. One standard alcoholic drink was one medium size glass of wine (120 mL), one Birille of Tej, one Tassa of Tella, one single measure of spirit/Areki Melekia (30 mL), or one 330 mL bottle of regular beer (29, 31).

Adults were classified as never-smokers (if they had never smoked a cigarette in their lives), previous smokers (if they had smoked previously but not in the previous month), and current smokers (if they had smoked cigarettes in the previous month) (29).

#### Data quality assurance

The investigators prepared the questionnaire in English, and then forward and backward translations into Amharic were done by English and Amharic-versed individuals to ensure consistency. The primary investigator provided training to the data collectors and supervisor. Before the actual data collection, the questionnaire was pretested on 5% of the study subjects to assess its clarity and sociocultural compatibility. The results of the pretest were not used in the final study. Every stage of the data collection process was checked for accuracy, completeness, and consistency.

#### Data processing and analysis

The data were inspected visually for completeness, and the responses were coded and entered into Epi-data version 4.6, of which 10% of the responses were chosen randomly and examined for data entry consistency. Then, data were exported to SPSS version 25 for further analysis. For continuous variables, summary measures were used, whereas percentages and frequencies were used for categorical variables. The percentage of those with controlled blood pressure in each group was calculated. The most likely demographic-, behavioral-, lifestyle-, clinical-, and medication-related variables that have a known association with blood pressure control were compared at baseline between statin user and non-user hypertensive patients to see the presence of any statistically significant difference. To see whether there was a statistically significant difference between the two groups, an independent sample *t*-test for normally distributed continuous variables and Pearson's chi-square (χ2-test) for categorical variables were used.

According to the AHA/ACC 2017 recommendations, patients with hypertension were further subdivided into either controlled or uncontrolled BP groups based on the attainment of blood pressure targets. The cut point used to demonstrate BP control was three months following the initial BP value record after starting treatment; because of reported clinical evidence that shows BP decreased substantially after 8–12 weeks of statin therapy (32, 33).

The two groups' mean reductions in DBP and SBP were compared. The baseline DBP and SBP were subtracted from the values of DBP and SBP at 3 and 6 months, and the difference was compared to see if there was an association between blood pressure reduction and statin use using an independent sample *t*-test. The odds ratio (OR) and 95%confidence interval (CI) for the association between blood pressure control and statin use were computed by binary logistic regression analysis. For adjusting any confounding effects, the variables that have significant differences (*P*-value of < 0.05) at baseline between the two groups were analyzed using a multivariable logistic regression model to see if the association exists after adjusting for those variables. In Model 1: age was included as a possible sociodemographic confounder variable; and in Model 2: the added presence of dyslipidemia and duration of antihypertensive treatment. All tests were two-sided and the odds ratio (OR) with a 95% confidence interval (CI) was calculated with a *P*-value of <0.05 as the cut point for establishing statistical significance association. To determine the model fit of each variable in a logistic regression model, the Hosmer and Lemeshow goodness-of-fit test was used. Homogeneity of variances was assumed for the variables included in the *t*-test.

### Ethical considerations

The study was carried out in line with the Helsinki Declaration. All participants were given written informed consent after the study's nature was properly explained verbally, and participation was voluntary. The Institutional Review Board of the University of Gondar College of medicine and health sciences granted ethical clearance with the reference number SOP/063/2020. The Felege Hiwot Comprehensive Specialized Hospital provided a permission letter to conduct the study. Furthermore, the confidentiality and privacy of the information obtained from the patient's records were actively protected.

## Results

### Study participants' enrollment and sociodemographic factors

**Figure 1:**
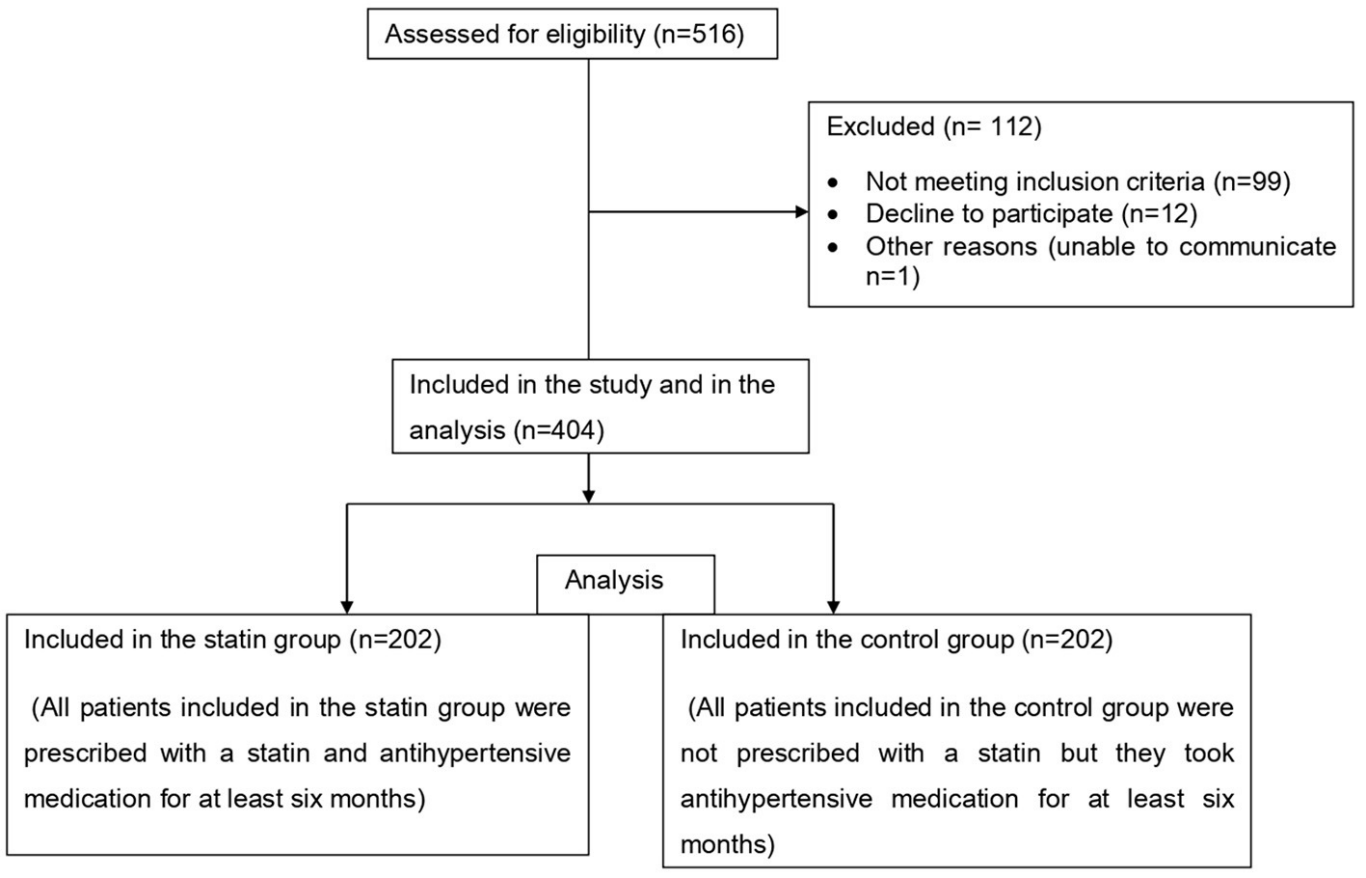
Participants' enrolment flowchart of patients with hypertension attending outpatient clinic at the Felege Hiwot Comprehensive and Specialized Hospital, northwest Ethiopia.

A total of 516 patients with hypertension were approached, all of whom had been on standard antihypertensive treatment for at least 6 months. Of this, 112 were excluded from the study, since they did not fulfill the inclusion criteria; and 404 patients were included in the final analysis after being evaluated for eligibility and consenting to participate. The statin group consisted of 202 patients (50%) who had been prescribed a statin for at least 6 months, while the control group (non-statin user group) consisted of the remaining 202 patients who had not been administered a statin (Figure 1).

The mean age of respondents was 59.8 ± 10.1 years and around one-fourth of them (25.5%) were above 65 years of age. More than one-third (37.9%) of the patients were women and nearly two-thirds (63.6%) were married; and 66 (16.3%) of the patients were rural residents.

There were no significant differences between patients in the control group and statin group regarding gender (41.6% male vs. 34.2% female participants; *P* = 0.124), current body mass index (mean 23.4 ± 3.3 kg/m^2^ vs. 23.3 ± 3.0 kg/m2, *P* = 0.571), and place of residence (18.3% urban area vs. 14.3%; *P* = 0.282), respectively.

Likewise, the proportion of patients regarding education level, marital status, religion, and type of occupation did not significantly differ in the two groups.

However, there was a statistically significant age difference between the groups. Statin users were found to be older than non-users (the mean age for statin users was 60.8 ± 10.4 years vs. 58.7 ± 9.8 years for non-statin users; *P* = 0.032) (Table 1).

**Table 1:** Sociodemographic features of patients with hypertension attending the outpatient clinic at FHCSH, northwest Ethiopia.

**Sociodemographic variables**		**Total participants (*****n*** = **404)**		**Statin users (*****n*** = **202)**		**Statin non-users (*****n*** = **202)**		**X^2^^−*Test*^**	***P*-value**
		**Frequency**	**%**	**Frequency**	**%**	**Frequency**	**%**		
Sex	Male	251	62.1	133	65.8	118	58.4	2.367	0.124
	Female	153	37.9	69	34.2	84	41.6		
Age (mean ± SD)^*^		59.8 ± 10.1		60.8 ±10.4		58.7 ± 9.8		2.163	0.032
BMI (mean ± SD)^*^		23.3 ± 3.1		23.2 ± 3.0		23.4 ± 3.3		0.502	0.571
Education	Illiterate	125	30.9	57	28.2	68	33.7	4.457	0.216
	Primary education	99	24.5	45	22.3	54	26.7		
	Secondary education	103	25.5	55	27.2	48	23.8		
	Collage/university and above	77	19.1	45	22.3	32	15.8		
Marriage	Married	257	63.6	136	67.3	121	59.9	3.371	0.338
	Single	68	16.8	28	13.9	40	19.8		
	Divorced	46	11.4	21	10.4	25	12.4		
	Widowed	33	8.2	17	8.4	16	7.9		
Religion	Orthodox	341	84.4	168	83.1	173	85.6	2.002	0.368
	Muslim	44	10.9	26	12.9	18	9.0		
	Protestant	19	4.7	8	4.0	11	5.4		
Residence	Rural	66	16.3	29	14.3	37	18.3	1.159	0.282
	Urban	338	83.7	173	85.7	165	81.7		
Occupation	Farmer	54	13.4	21	10.4	33	16.3	8.545	0.129
	Civil servant	99	24.5	46	22.8	53	26.2		
	Housewife	66	16.3	29	14.3	37	18.3		
	Private worker	107	26.5	59	29.2	48	23.8		
	Retired	30	7.4	18	9.0	12	6.0		
	Other^**^	48	11.9	29	14.3	19	9.4		

* Age and BMI are continuous variables expressed as mean ± SD and the *p*-value was computed with an independent *t*-test.

** Other occupation includes student, merchant, and carpenter.

### Study participants' behavioral/lifestyle characteristics

Of the 404 subjects, only 11 (2.7%) were current smokers, 85 (21%) were not adherent to their medications, and the majority of the respondents (75.2%) were physically inactive. Physically inactive participants predominated, but there was no difference in their distribution between the two groups. There was no statistically significant variation in patients in the control group vs. statin group regarding physical activity since hypertension diagnosis (physically inactive (73.3 vs. 77.2%), insufficiently physically active (22.3 vs. 14.9%), and physically active (4.4 vs. 7.9%), *P* = 0.075), medication adherence since diagnosis (nonadherent; 22.8 vs. 19.3%; *P* = 0.393), and adding salt in food (rarely; 43.0 vs. 53.5%; *P* = 0.126).

Similarly, patients' proportions regarding smoking status after diagnosis, alcohol drinking, consumption of fruit, vegetables, and grains, and high saturated fat consumption did not differ significantly between the two groups (Table 2).

**Table 2:** Lifestyle/behavioral features of patients with hypertension attending the outpatient clinic at FHCSH, northwest Ethiopia.

**Base line life style behavior variables**		**Statin users (*****n*** = **202)**		**Statin non-users (*****n*** = **202)**		**X^2 − Test^**	***p*-value**
		**Frequency**	**%**	**Frequency**	**%**		
Physical activity status after diagnosis	Inactive	156	77.2	148	73.3	5.171	0.075
	Insufficient	30	14.9	45	22.3		
	Active	16	7.9	9	4.4		
Alcohol drinking status since diagnosis	None	148	73.2	159	78.7	2.737	0.255
	Moderate	50	24.8	37	18.3		
	Excessive	4	2.0	6	3.0		
Smoking status after diagnosis	Never smoked	178	88.1	189	93.6	3.609	0.165
	Former smoker	17	8.4	9	4.4		
	Current smoker	7	3.5	4	2.0		
Fruits, vegetables, and grains included in diet after diagnosis	Never	27	13.4	16	7.9	7.423	0.060
	Rarely	144	71.2	154	76.2		
	Usually	19	9.4	27	13.4		
	Always	12	6.0	5	2.5		
Consumption of foods with high saturated fat	Never	181	89.6	190	94.0	2.673	0.102
	Rarely	21	10.4	12	6.0		
Added salt in food	Never	82	40.5	95	47.0	5.716	0.126
	Rarely	108	53.5	87	43.0		
	Usually	5	2.5	11	5.6		
	Always	7	3.5	9	4.4		
Medication adherence	Good	163	80.7	156	77.2	0.730	0.393
	Poor	39	19.3	46	22.8		

### Study participants' clinical characteristics

Out of the total respondents, 67 (16.6%) have reported a family history of high BP, whereas 61.4% had another comorbidity, with 98 (24.3%) having diabetes mellitus and 23.8% having cardiovascular disease. There were no differences which were significant between patients in the control group and statin group regarding family history of high BP (have a family history of high BP; 19.3 vs. 13.8%; *P* = 0.141), frequency of antihypertensive drug use per day (once a day 54.9 vs. 62.9%; *P* = 0.106), and presence of diabetes mellitus (Yes; 42.7 vs.36.6%; *P* = 0.327), respectively.

Furthermore, the patients' proportion with cardiovascular diseases, chronic kidney disease, asthma, and HIV/AIDS did not differ significantly in the two groups. On the other hand, dyslipidemia as comorbidity increased remarkably in the statin group (64.1 vs. 1.7% *P* < 0.001). Moreover, the mean duration of antihypertensive medication treatment was higher significantly in the statin user group (4.2 ± 2.0 vs. 3.7 ± 1.7 years, *P* = 0.011), which could be a confounding variable (Table 3).

**Table 3:** Clinical features of patients with hypertension attending the outpatient clinic at FHCSH, northwest Ethiopia.

**Variables**		**Statin users (*****n*** = **202)**		**Statin non-users (*****n*** = **202)**		**X^2 − Test^**	***p*-value**
		**Frequency**	**%**	**Frequency**	**%**		
Family history of high BP	Yes	28	13.8	39	19.3	2.165	0.141
	No	174	86.2	163	80.7		
Stage of HTN at diagnosis	Stage I	98	48.5	82	40.6	2.565	0.109
	Stage II	104	51.5	120	59.4		
Duration antihypertensive drug treatment^*^		4.2 ± 1.9		3.7 ± 1.7		2.560	0.011
Frequency of Health follow up	Monthly	34	16.8	24	11.9	2.013	0.156
	Every 3 months	168	83.2	178	89.1		
Presence of comorbidities	Yes	131	64.9	117	58.0	2.047	0.153
	No	71	35.1	85	42.0		
Diabetes mellitus	Yes	48	36.6	50	42.7	0.960	0.327
	No	83	63.4	67	57.3		
Chronic kidney disease	Yes	8	6.1	4	3.4	0.970	0.325
	No	123	93.9	113	96.6		
Cardiovascular diseases	Yes	52	39.7	44	37.6	0.114	0.736
	No	79	60.3	73	52.4		
Asthma	Yes	3	2.3	8	6.8	3.015	0.082
	No	128	97.7	109	93.2		
HIV/AIDS	Yes	7	5.3	6	5.1	0.006	0.939
	No	124	94.7	111	94.9		
Dyslipidemia	Yes	84	64.1	2	1.7	106.27	0.000
	No	47	35.9	115	98.3		
Other^**^	Yes	3	2.3	7	6.0	2.178	0.140

* Duration of antihypertensive drug treatment was computed using the independent sample *t*-test and expressed as (Mean ± SD).

** Other comorbidities include gout, peptic ulcer disease, and depression.

### Medication-related features of the study participants

The groups did not differ significantly in terms of the number and type of antihypertensive drugs used. Enalapril was the most frequently prescribed drug among both groups (52.2%) followed by hydrochlorothiazide (42.5%). There was no significant difference between patients in the control group and statin group regarding the frequency of antihypertensive drug use per day (once a day antihypertensive use; 54.9 vs. 62.9%, *P* = 0.106) and the number of antihypertensive drug/s the patient is using (dual therapy; 33.1 vs. 27.2% *P* = 0.388).

In terms of specific antihypertensive drug use and the presence of medications other than antihypertensive therapy, there was no significant difference. Among statin users, two types of statins were used, atorvastatin and simvastatin; a quarter of statin users 52 (25.2%) take simvastatin and the rest were using atorvastatin (Table 4).

**Table 4:** Medications-related characteristics of patients with hypertension attending the outpatient clinic at FHCSH, northwest Ethiopia.

**Variables**			**Statin users (*****n*** = **202)**		**Statin non-users (*****n*** = **202)**		**X^2^ Test**	***p*-value**
			**Frequency**	**%**	**Frequency**	**%**		
Frequency of anti-HTN drug use per day		Once	127	62.9	111	54.9	2.618	0.106
		Twice	75	37.1	91	45.1		
Number of anti-HTN drug/s the patient is using		Mono-therapy	130	64.4	117	57.9	1.893	0.388
		Dual therapy	55	27.2	67	33.1		
		Triple therapy	17	8.4	18	9.0		
Name of antihypertensive	Hydrocloroizide	Yes	80	39.6	92	45.5	1.458	0.227
		No	122	60.4	110	54.5
	Enalapril	Yes	111	55.0	96	47.5	2.229	0.135
		No	91	45.0	106	52.5
	Nifedepine	Yes	42	20.8	54	26.7	1.968	0.161
		No	160	79.2	148	73.3
	Amlodipine	Yes	39	19.3	49	24.3	1.453	0.228
		No	163	80.7	153	76.7
	Atenolol	Yes	13	6.4	7	3.5	1.894	0.169
		No	189	93.6	195	96.5
	Other anti-HTN drug use^*^	Yes	2	1.0	4	2.0	0.677	0.411
Presence of medications other than antihypertensive drugs	Metformin	Yes	35	17.3	34	16.8	2.627	0.105
	Glibenclamide	Yes	9	4.4	13	6.4	0.41	0.839
	NSAIDs	Yes	14	6.9	20	10.0	0.051	0.821
	Insulin	Yes	13	6.4	13	6.4	0.611	0.435
	Clopidogrel	Yes	5	2.4	8	3.9	0.113	0.736
	ART	Yes	7	3.4	6	2.9	0.677	0.411
	Other medications^**^	Yes	34	16.8	35	17.3	1.741	0.187

* Other anti-HTN drug includes furosemide and spironolactone.

** Other medications include digoxin, warfarin, allopurinol, omeprazole, and fluoxetine.

### Blood pressure control status of the study participants

Even though there was no statistically significant difference in baseline mean SBP and DBP between statin users (164.5 ± 14.0 vs. 162.3 ± 12.6, *P* = 0.107) and statin non-users (100.6 ± 8.7 vs. 99.8 ± 7.7, *P* = 0.317), BP control based on the ACC/AHA 2017 guideline was higher significantly (*P* = 0.022) in the statin plus antihypertensive drug group (52.5%) than in antihypertensive drug alone group (41.0%) after 3 months of treatment.

Also, isolated SBP and DBP controls were higher among statin users than in the control group (52.5% for systolic blood pressure control vs. 41.6%, *P* = 0.028 and 60.9% for isolated diastolic control vs. 47.0%, *P* = 0.005) (Table 5).

**Table 5:** Blood pressure control status of patients with hypertension attending the outpatient clinic at FHCSH, northwest Ethiopia.

**Variables**		**Statin users (*****n*** = **202)**		**Statin non-users (*****n*** = **202)**		**X^2^ Test**	***p*-value**
		**Frequency**	**%**	**Frequency**	**%**		
Systolic BP controlled	Yes	106	52.5	84	41.6	4.809	0.028
	No	96	47.5	118	58.4		
Diastolic BP controlled	Yes	123	60.9	95	47.0	7.811	0.005
	No	79	39.1	107	53.0		
Both the systolic and diastolic BP	Yes	106	52.5	83	41.0	5.259	0.022
controlled	No	96	47.5	119	59.0		

### Mean blood pressure reduction status of the study participants

Accordingly, significantly higher mean systolic BP reduction from baseline (30.6 mmHg ± 18.7 vs. 25.24 mmHg ± 13.9 *P* = 0.001) and mean DBP reduction (20.4 mmHg ± 11.3 vs. 17.2 ± 9.0 *P* = 0.002) were observed in the statin user group after 3 months of treatment compared to their counterparts (Table 6).

**Table 6:** *T*-test of difference in mean change of SBP and DBP of patients with hypertension attending the outpatient clinic at FHCSH, northwest Ethiopia.

**Variables**	**Statin users (*n* = 202)**	**Statin non-users (*n* = 202)**	***T*-test**	***P*-value**
	**Mean** ±**SD**	**Mean** ±**SD**		
SBP at initiation	164.5 ± 14.0	162.3 ± 12.6	1.61	0.107
SBP after 3 months	133.8 ± 16.4	137.1 ± 16.1	2.01	0.044
SBP after 6 months	131.5 ± 15.2	134.6 ± 14.3	2.08	0.037
DBP at initiation	100.6 ± 8.7	99.84 ± 7.7	1.00	0.317
DBP after 3 months	80.1 ± 8.3	82.6 ± 8.7	2.83	0.005
DBP after 6 months	79.3 ± 7.9	81.6 ± 8.4	2.77	0.006
Mean d/f b/n SBP at initiation and at 3 months^a^	30.6 ± 18.7	25.2 ± 13.6	3.30	0.001
Mean d/f b/n SBP at initiation and at 6 months^b^	32.9 ± 18.2	27.7 ± 14.0	3.22	0.001
Mean d/f b/n DBP at initiation and at 3 months^c^	20.4 ± 11.3	17.2 ± 9.0	3.17	0.002
Mean d/f b/n DBP at initiation and at 6 months^d^	21.2 ± 11.0	18.1 ± 9.1	3.06	0.002

a Mean difference between systolic blood pressures at initiation and at 3 months.

b Mean difference between systolic blood pressures at initiation and at 6 months.

c Mean difference between diastolic blood pressures at initiation and at 3 months.

d Mean difference between diastolic blood pressures at initiation and at 6 months.

**Figure 2:**
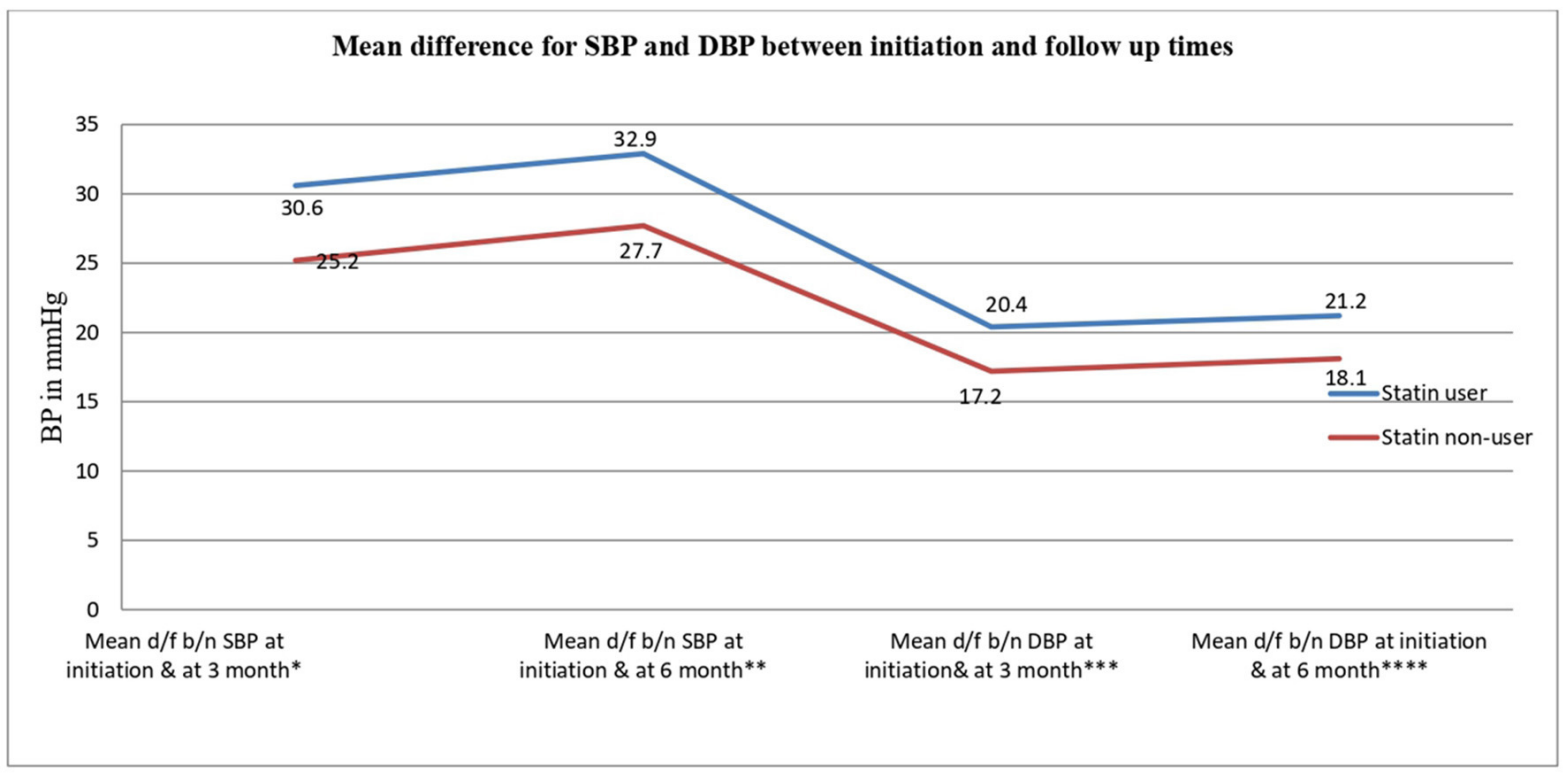
Line graph showing the trend of blood pressure mean differences at various times between statin user and non-user hypertensive outpatients at the Felege Hiwot Comprehensive and Specialized Hospital, northwest Ethiopia. ^*^Mean difference between systolic blood pressures at initiation and at 3 months. ^**^Mean difference between systolic blood pressures at initiation and at 6 months. ^***^Mean difference between diastolic blood pressures at initiation and at 3 months. ^****^Mean difference between diastolic blood pressures at initiation and at 6 months.

Similarly, the mean systolic and diastolic BP reduction after 6 months of observation from baseline resulted in a higher and more significant reduction among statin users when compared to the control group (32.9 mmHg ± 18.7 vs. 27.7 ± 14.0 *P* = 0.001 and 21.2 mmHg ± 11.0 vs. 18.1 ± 9.1 *P* = 0.002) (Figure 2).

The use of statin therapy was associated with a higher mean SBP reduction of 5.4 mmHg (95% CI, 2.2 to 8.6, *P* = 0.001) and mean DBP reduction of 3.2 mmHg (95% CI, 1.2 to 5.2 *P* = 0.002) after 3 months of treatment from baseline; and mean SBP reduction of 5.2 mmHg (95% CI, 2.0 to 8.4 *P* = 0.001) and mean DBP reduction of 3.1 mmHg (95% CI, 1.1 to 5.0 *P* = 0.002) after 6 months of treatment from baseline (Table 7).

**Table 7:** *T*-test result for statin users versus non-users in terms of BP reduction from baseline (pre-drug BP value) of patients with hypertension attending the outpatient clinic at FHCSH, northwest Ethiopia.

**Variables**	**At three month**			**At 6 month**		
	**Mean**	**95% CI**	***P*** **-value**	**Mean**	**95% CI**	***P*** **-value**
Mean difference of Mean SBP reduction (mmHg) for statin users when compared to non-users	5.4	2.2–8.6	0.001	5.2	2.0–8.4	0.001
Mean difference of Mean DBP reduction (mmHg) for statin users when compared to non-users	3.2	1.2–5.2	0.002	3.1	1.1–5.0	0.002

### Logistic regression for blood pressure control and statin use

Because age, the presence of dyslipidemia as comorbidity, and the mean duration of antihypertensive drug treatment were all significantly higher in the statin user group, these variables were integrated into a logistic regression model to adjust the OR of controlled blood pressure associated with statin therapy.

The crude model in Table 8 reveals that statin therapy enhances the likelihood of having blood pressure controlled 1.58 times [OR 1.58; 95% CI, 1.068–2.346]. After controlling for age in Model 1, the odds of having controlled blood pressure were 1.6 times [95% CI, 1.106–2.451] higher among statin users as compared to their counterparts. This likelihood remained unchanged when adding the presence of dyslipidemia as comorbidity and the mean duration of antihypertensive treatment in Model 2 which were statistically significantly different at baseline between the two groups. Still, statin therapy increased the odds of having controlled blood pressure by a factor of 5.98 [OR 5.986; 95% CI 2.773–12.922] when compared to statin non-users indicating that the use of statins among patients with hypertension was significantly associated with controlled blood pressure.

**Table 8:** The association of statin use with controlled BP in bivariable and multivariable binary logistic regression analyses among patients with hypertension attending the outpatient clinic at FHCSH, northwest Ethiopia.

**Model type**	**Adjusted OR**	**AOR 95% CI**	***P*-value**
Mode 0: unadjusted	1.583^*^	1.068–2.346	0.022
Model 1: controlling for age	1.646	1.106–2.451	0.014
Model 2: controlling for age, presence of dyslipidemia, and duration of antihypertensive therapy	5.986	2.773–12.922	<0.001

* Crude odds ratio.

The bivariable and multivariable binary logistic regression analyses show age, presence of dyslipidemia, and duration of antihypertensive therapy affect the association of BP control with statin therapy.

## Discussion

The study was intended to investigate the association between the use of statin therapy and BP control in patients with hypertension. This study revealed that after 3 months of treatment with statins, BP control based on the ACC/AHA 2017 guideline was significantly higher (*P* = 0.022) in the statin group (52.5%) in comparison to the control group (41.0%). This finding was similar to previous studies (14, 16, 27). In this study, the proportion of patients concerning gender, presence of CKD, diabetes, and the class and number of antihypertensive medications used did not significantly differ in both groups; and this is consistent with other studies conducted in the United States of America and Portugal (16, 26).

In this study, statin users were on average 2 years older than non-users; this result is in agreement with previous studies (14, 16, 24). This may be due to the possibility that aging increases the burden of atherosclerosis and other cardiovascular conditions; as a result, elderly people have a higher risk of cardiovascular mortality and morbidity than younger people, and they require more rigorous treatment of modifiable risk factors including dyslipidemia, which may necessitate statin therapy (34). Antihypertensive drug therapy mean duration was also longer among statin users which is in agreement with a study by Morgado and associates (16). This may be due to the high probability of statin user hypertensive patients being aged with different comorbidity that may increase the need to use statins as primary and secondary cardiovascular protection.

In the current study, even though there was no difference which was significant in baseline mean SBP and DBP between the two groups after 3 months of statin treatment, blood pressure control (<130/80 mmHg) was higher significantly (*P* = 0.022) in the statin user group (52.5%) than in the control group (41.0%). This is in line with several studies that show statins' ability in reducing significantly the DBP and SBP, beyond their lipid-lowering properties (14–17, 24–27, 35).

The result in this study of superior BP control in patients with hypertension taking a prescribed statin supports the theory that statins may also have an antihypertensive effect (27, 36, 37). A study conducted in the United States of America found that when compared to people who did not use statins, more statin users had significantly controlled their blood pressure (52.2 vs. 38.0%) (27). This could be explained by statins' pleiotropic effects such as decreased blood pressure, which go beyond cholesterol reduction which in turn has overwhelming benefits in preventing cardiovascular events (12).

The current study indicates that using a statin improves the likelihood of having blood pressure controlled by 1.58 times [OR 1.58; 95% CI, 1.068–2.346]. After adjusting for age, the presence of dyslipidemia as a comorbidity, and the mean duration of antihypertensive treatment which were statistically significantly different at baseline in the two groups, still using statin increased the odds of controlled blood pressure 5.98 times [OR 5.986; 95% CI, 2.773–12.922]. After controlling for potential confounding factors, the relationship was still maintained. This is in line with a previous study done in the United States of America that showed after controlling for demographic factors, statin users were two times (95% CI, 1.46–2.72) more likely than non-users to have their blood pressure under control (140/90 mmHg). In the study, the likelihood of having controlled blood pressure remained more likely among statin users (OR 1.46, 95% CI, 1.05–2.05) after further adjusting for diabetes, BMI, exercise, smoking, antihypertensive medications, and low-salt diet (27).

Adding to the growing body of evidence that statin medication can help with blood pressure control, a study in Portugal indicated that statin therapy enhances the likelihood of having blood pressure under control [OR 4.46; 95% CI, 1.64–12.15]. The study found the same statistically significant relationship after controlling for the length of antihypertensive treatment [OR 5.23; 95% CI 1.86–14.67] (16).

The physiologic effects of statins on the body, which point to the “pleiotropic” effects of statins, such as anti-inflammatory effects, improved endothelial function, stabilization of atherosclerotic plaques, antioxidant properties, and increased nitric oxide (NO) bioavailability, are possible explanations for this association (38). Effects on the endothelial vaso-reactivity or renin-angiotensin system could also explain association (36). Statins, on top of their undeniable potential to lower lipid profile, have many other biological effects, mostly related to improving arterial compliance and endothelial function. As shown in Figure 2, the mean systolic and diastolic BP reduction after 6 months of observation from baseline resulted in a higher and more significant reduction among statin users when compared to the control group (32.9 mmHg ± 18.7 vs. 27.7 ± 14.0, *P* = 0.001 and 21.2 mmHg ± 11.0 vs. 18.1 ± 9.1, *P* = 0.002). This blood pressure control gap between statin users and non-users over time can be explained by the possible additive effect with antihypertensive and statin drugs on better BP control when used together, which was supported by former studies that found BP can be controlled much better with a combination of statins and antihypertensive medications than with either treatment alone (39–41).

In this retrospective cohort study, the use of statin therapy was associated with not only enhanced BP control but also a higher mean SBP reduction of 5.4 mmHg (95% CI, 2.2 to 8.6, *P* = 0.001) and mean DBP reduction of 3.2 mmHg (95% CI, 1.2 to 5.2 *P* = 0.002) after 3 months of treatment from baseline; and mean SBP reduction of 5.2 mmHg (95% CI, 2.0 to 8.4, *P* = 0.001) and mean DBP reduction of 3.1 mmHg (95% CI, 1.1 to 5.0, *P* = 0.002) after 6 months of treatment from baseline was observed with it.

In line with this result, in a randomized, double-blind study, Ferrier et al. reported a mean reduction of −6 mmHg in SBP after 3 months of atorvastatin treatment in a sample of patients with isolated systolic hypertension (33). Another study also demonstrated that statins reduced SBP by 3.3 mmHg and DBP by an average of 1.9 mmHg (*P* < 0.01) among antihypertensive drug users (*P* = 0.02).

The mean SBP decrement of 5.4 mmHg and mean DBP decrement of 3.2 mmHg after 3 months of treatment from baseline is in agreement with data obtained from a study by Kuklinska and associates, that used patients with normolipid who were taking standard HTN treatment; although both groups' baseline BP scores were similar, after 3 months of atorvastatin therapy, the mean changes in SBP and DBP were 5.7 mmHg (95% CI, 4.1 to 7.2 mmHg) and 3.9 mmHg (95% CI, 2.7 to 5.0 mmHg), respectively. This finding indicates the presence of a significant association with atorvastatin use for blood pressure control (42). In addition, Morgado and associates report significantly lower SBP and DBP (−6.7 mmHg, *P* = 0.020 and −6.4 mmHg, *P* = 0.002) levels, respectively, in the statin user group (16). Similar to this study, a retrospective study conducted in Italy in 2017 demonstrated that the use of statins was linked with the independent and strongest association with 24 h and night-time BP control, even after controlling for sex, BMI, age, number of antihypertensive drugs, and diabetes (model 1), or the presence/absence of antihypertensive therapy (17). This favorable effect of statins on BP control could also be explained by the positive behavior of patients who are on lipid-lowering therapy regarding cardiovascular prevention strategies; as indicated by former studies that show patients who use lipid-lowering therapy had good treatment persistence and adherence (43, 44).

Statins are likely to benefit patients with uncontrolled BP whose modulation of vascular resistance and peripheral vascular tone is significantly compromised (40, 45). In addition to this, because of the physiologic effect they share, lipid-lowering therapies other than statins, such as nicotinic acid (niacin), omega-3 fatty acids, and fatty acid esters and fibric acid derivatives (Fenofibrate), have also a favorable effect on reducing and controlling blood pressure (46–50).

Conversely from the above findings, some studies show contradiction about the association of BP control and reduction with statin use. Some earlier studies found no BP-lowering impact of statins in patients with normotension and well-controlled hypertension (32, 39). A meta-analysis of 936 patients with hypertension and 4,692 patients with normotension found that statin therapy does not result in a substantial decrease in systolic or diastolic BP in either patients with normotension or hypertension (39). This may be due to high heterogeneity between studies. Another reason for this disparity could be that the participants were predominantly normotensive, which could reduce the statins benefit because the possible hypotensive effect of statins has been hypothesized to be more evident in patients with higher baseline blood pressure (20, 26, 40, 51). Furthermore, in the presence of comorbid conditions, there may be drug—drug interactions between the antihypertensive medicine and the other medications being used, which might potentially antagonize the antihypertensive medication's therapeutic effect and result in uncontrolled BP.

This study found that when antihypertensive and statin drugs were used together, they had an additive effect for better BP control, which was supported by former studies that found BP can be controlled much better with a combination of statins and antihypertensive medications than with either treatment alone (39, 41).

This could be because of a possible additive effect of these medications. The conflicting findings of some studies indicate that this topic is not fully resolved and that more research is needed.

### Limitations of the study

Due to the retrospective nature of the study, blood pressure values were taken as documented in the patients' medical charts, which reflected actual clinical practice; nonetheless, these values may be subjected to measurement and recording errors. Furthermore, some issues were not explored in this study and will necessitate additional study designs. These include the impact of certain combinations of antihypertensive drugs and statins and the impact of various statin regimens and dosages. The authors strongly encourage future researchers to use prospective study designs to address the issues that were not covered in this study.

## Conclusion

This study concluded that the use of statins is associated with BP control (<130/80 mmHg) among patients with hypertension in a real-world clinical setting. The current study found that, in patients with hypertension who need to take a statin concurrently, the use of a statin can enhance blood pressure control and decrease 5.4 and 3.2 mmHg of SBP and DBP, respectively, after 3 months of treatment. This positive blood pressure control and reduction may decrease the number of antihypertensive drugs and doses needed to achieve satisfactory BP control, which could have some therapeutic implications. The results of this study may have important implications for the safe and effective prevention of cardiovascular diseases, especially in patients with hypertension whose blood pressure is not adequately controlled by antihypertensive treatment alone.

## Data availability statement

The original contributions presented in the study are included in the article/supplementary material, further inquiries can be directed to the corresponding author.

## Ethics statement

The studies involving human participants were reviewed and approved by the Institutional Review Board of the University of Gondar's School of Pharmacy, who approved this study under the reference number SOPS 063/2020. The patients/participants provided their written informed consent to participate in this study.

## Author contributions

RA, SK, and MB conceived the study and were involved in its design, coordination, and review of the article, analysis, report writing, and manuscript preparation. The final manuscript was read and approved by all authors.
